# A novel inhalable quercetin-alginate nanogel as a promising therapy for acute lung injury

**DOI:** 10.1186/s12951-022-01452-3

**Published:** 2022-06-11

**Authors:** Yi-Bing Chen, Ya-Bin Zhang, Yu-Le Wang, Prabhleen Kaur, Bo-Guang Yang, Yan Zhu, Lei Ye, Yuan-Lu Cui

**Affiliations:** 1 State Key Laboratory of Component-based Chinese Medicine, Research Center of Traditional Chinese Medicine, Tianjin University of Traditional Chinese Medicine, No. 10 Poyanghu Road, West District of Tuanbo New Town, Jinghai District, Tianjin, 301617 China; 2 Department of Pharmaceutics, Key Laboratory of Chemical Biology (Ministry of Education), School of Pharmaceutical Sciences, Shandong University, Jinan, 250012 Shandong China; 3 First Teaching Hospital of Tianjin University of Traditional Chinese Medicine, 300381 Tianjin, China; 4 National Clinical Research Center for Chinese Medicine Acupuncture and Moxibustion, 300381 Tianjin, China; 5 Shandong Provincial Key Laboratory of Fluorine Chemistry and Chemical Materials, School of Chemistry and Chemical Engineering, University of Jinan, 250022 Jinan, China; 6 Research and Development Center of TCM, Tianjin International Joint Academy of Biotechnology and Medicine, TEDA, 300457 Tianjin, China; 7 Department of Chemical Engineering, University of Washington, Seattle, WA 98195 USA; 8 Department of Biomedical Engineering, The Chinese University of Hong Kong, Hong Kong, China; 9 Pharmaceutical Informatics Institute, College of Pharmaceutical Sciences, Zhejiang University, Hangzhou, China

**Keywords:** COVID-19, Acute lung injury, Quercetin, Nanogel, Ultrasonic aerosol inhalation

## Abstract

**Background:**

Acute lung injury (ALI), a severe health-threatening disease, has a risk of causing chronic pulmonary fibrosis. Informative and powerful evidence suggests that inflammation and oxidative stress play a central role in the pathogenesis of ALI. Quercetin is well recognized for its excellent antioxidant and anti-inflammatory properties, which showed great potential for ALI treatment. However, the application of quercetin is often hindered by its low solubility and bioavailability. Therefore, to overcome these challenges, an inhalable quercetin-alginate nanogel (QU-Nanogel) was fabricated, and by this special “material-drug” structure, the solubility and bioavailability of quercetin were significantly enhanced, which could further increase the activity of quercetin and provide a promising therapy for ALI.

**Results:**

QU-Nanogel is a novel alginate and quercetin based “material-drug” structural inhalable nanogel, in which quercetin was stabilized by hydrogen bonding to obtain a “co-construct” water-soluble nanogel system, showing antioxidant and anti-inflammatory properties. QU-Nanogel has an even distribution in size of less than 100 nm and good biocompatibility, which shows a stronger protective and antioxidant effect in vitro. Tissue distribution results provided evidence that the QU-Nanogel by ultrasonic aerosol inhalation is a feasible approach to targeted pulmonary drug delivery. Moreover, QU-Nanogel was remarkably reversed ALI rats by relieving oxidative stress damage and acting the down-regulation effects of mRNA and protein expression of inflammation cytokines via ultrasonic aerosol inhalation administration.

**Conclusions:**

In the ALI rat model, this novel nanogel showed an excellent therapeutic effect by ultrasonic aerosol inhalation administration by protecting and reducing pulmonary inflammation, thereby preventing subsequent pulmonary fibrosis. This work demonstrates that this inhalable QU-Nanogel may function as a promising drug delivery strategy in treating ALI.

**Graphical Abstract:**

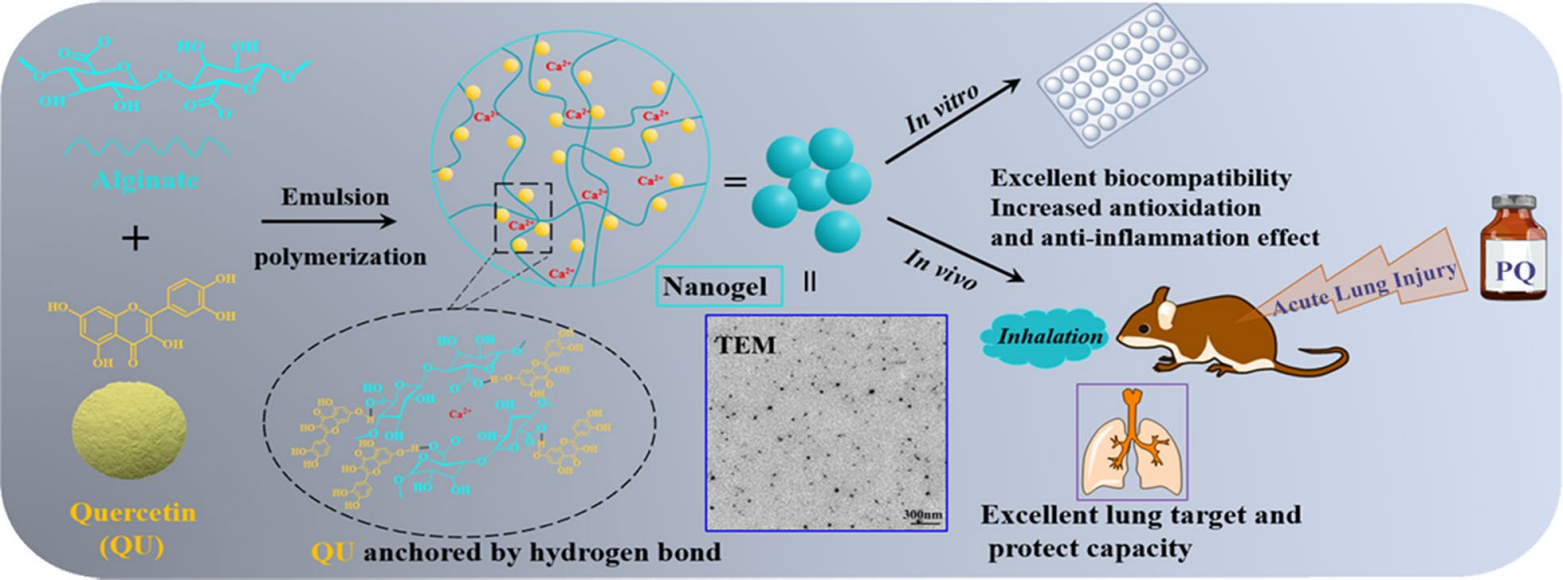

**Supplementary Information:**

The online version contains supplementary material available at 10.1186/s12951-022-01452-3.

## Highlights

- Quercetin (QU)-Nanogel shows a significant therapeutic effect on acute lung injury.
- Quercetin as an active substance, was also involved in the nanogel construction.
- The novel nanogel increase the bioavailability of quercetin.
- Inhalation of QU-Nanogel allows the drug to reach the lungs directly.

**Supplementary Information:**

The online version contains supplementary material available at 10.1186/s12951-022-01452-3.

## Background

Acute lung injury (ALI) can be caused by multiple pathological factors in clinical, which could further lead to respiratory distress and eventually develop into pulmonary fibrosis and chronic impairment of lung function [1, 2]. In the backdrop of the novel coronavirus pneumonia (COVID-19) global pandemic, especially for moderate and severe cases, ALI remains the most common symptom [3–5]. Therefore, the effective therapeutic method in ALI is also helpful for the treatment of COVID-19 [6]. The clinical features of ALI include the tissue infiltration of inflammatory cells, pulmonary edema, and arterial hypoxemia, which damage the vascular endothelium and alveolar epithelium, thus diminishing lung function. And high levels of epithelial apoptosis are critical in the pathogenesis of ALI [7, 8]. Since there is a lack of effective treatments in clinical [9], the main solution method for ALI is decreasing the absorption or decreasing the oxidant-induced cellular damage [10]. Thus, drugs with powerful antioxidant ability are a potential candidate for ALI treatment.

Flavonoids have strong antioxidant property than the most compounds, and quercetin is one of the most powerful antioxidants in flavonoids and widely exists in nature [11, 12]. In recent years, quercetin has attracted increasing attention due to its anti-inflammatory and anti-oxidation effects in cell models [13, 14]. In another report, quercetin showed a detoxification effect on paraquat (PQ) caused ALI rat model [15]. It can suppress the ongoing oxidative stress, acute inflammation, and cytokine storm, which can increase animal survival rate, improve lung edema and pathological symptoms [16, 17]. However, the low solubility and bioavailability have limited its application [18–20]. Therefore, designing a suitable carrier to increase bioavailability is urgently needed. Alginate, an FDA-approved natural polysaccharide, has excellent biocompatibility and is widely used in the biomedical field [21–23]. In addition, alginate can form hydrogels in metal cation solutions under mild conditions, which was applied in the microcapsule or nanogel preparation to treat various diseases by conventional administration route [24–26]. However, alginate has rarely been studied in the field of ultrasonic aerosol inhalation formulation due to the technical limitations, which is hard to prepare uniform alginate-based nanoparticles fitting for ultrasonic aerosol inhalation. As in previous studies, we have improved the technology and prepared a glycyrrhizin alginate nanogel less than 100 nm, showing good biocompatibility as a drug delivery carrier [27].

With the developments in pharmaceutical science, the ultrasonic aerosol inhalation method has gained increased attention due to the advantages of rapid drug absorption, convenient administration, good patient compliance, and reduced damage by gastrointestinal enzymes [28]. Most importantly, this method can directly deliver drugs to the respiratory system that can avoid the liver first-pass effect. Therefore, ultrasonic aerosol inhalation is being extensively applied for lung, nasal and throat diseases treatment in clinical [29–31]. However, most ultrasonic aerosol inhalation was applied to deliver hydrophilic drugs, limiting its application for drugs with poor water solubility. Thus, it is important to come up with a suitable carrier for hydrophobic drugs.

To overcome these challenges and develop effective therapeutic strategy for ALI, a quercetin-nanogel (QU-Nanogel) was prepared using the emulsion polymerization method. In this way, alginate and quercetin form “material-drug” structural inhalable nanogel through Ca^2+^crosslinking, in which quercetin can participate in the formation of the alginate nanogel, stabilized by hydrogen bonding between molecules to obtain a “co-construct” water-soluble nanogel system. Through this strategy, the solubility and bioavailability of quercetin have improved. QU-Nanogel with small particle size showed antioxidant properties. In the ALI rat model, QU-Nanogel reduced pulmonary inflammation significantly and prevented the subsequent pulmonary fibrosis, which showed an excellent pulmonary protective efficacy.

## Materials and methods

### Materials

Quercetin dihydrate was obtained from Sangon Biological Engineering Technology & Services Co., Ltd. Sodium alginate, Span 80, Tween 80, DAPI and paraquat (methyl viologen dichloride) were purchased from Sigma-Aldrich Co. (St. Louis, USA). Calcium chloride was purchased from Tianjin Fengchuan chemical reagent technologies Co., Ltd. DPPH (1, 1-diphenyl-2-picrylhydrazyl) was obtained from Alfa Aesar (Ward Hill, MA, USA). The interleukin-1β (IL-1β), tumor necrosis factor-α (TNF-α), and interleukin-6 (IL-6) ELISA kits were purchased from Peprotech Inc. (Rocky Hill, New Jersey, USA). Antioxidant capacity assay kit with FRAP method, Malonaldehyde (MDA) and Catalase (CAT) kits, Reactive Oxygen Species (ROS) assay kit, DiI dye, DiO dye and DAPI dye were obtained from the Beyotime Biotechnology (Shanghai, China).

### Preparation of nanogel

The QU-Nanogel was prepared using the emulsion polymerization method, per a previously published paper in our lab [27]. Briefly, 150 mL liquid paraffin, 1.05 mL span 80 mL and 0.45 mL tween 80 were added to a three-necked flask and agitated at 500 rpm for 30 min to obtain the oil phase. An amount of 45 mL of 0.5% (v/w) sodium alginate was added to the oil phase at a dripping rate of 0.75 mL/min, as the water phase. Then, 15 mL of an alcoholic solution containing calcium chloride and quercetin was added to the mixture. The final system was centrifuged to collect samples, and the obtained nanogels were washed and lyophilized. The rhodamine B labeled alginate was prepared per the literature [32]. Blank nanogel (BLK-Nanogel) was prepared with pure calcium chloride solution, using the same method. RBITC-labeled QU-Nanogel (RBITC-QU-Nanogel) were prepared with rhodamine B isothiocyanate labeled alginate, using the same method.

### Drug loading and encapsulation efficiency of QU-Nanogel

The drug loading and encapsulation efficiency were calculated using the following method. An amount of 25 mg of lyophilized QU-Nanogel was accurately weighed and resuspended in methanol and extracted under ultra-sonication for three divided times. The extraction solution was transferred to a 25 mL volumetric flask and bring to volume by methanol. The supernatant was then transferred to a volumetric flask and the precipitate was extracted three times. The absorbance of the supernatant was assayed using the HPLC method (Waters 2695, Milford, MA, USA). The conditions are described in the Additional file 1: S1 Condition for HPLC test. Encapsulation efficiency was measured using ultrafiltration method. Briefly, 10 mg QU-Nanogel was dissolved in 1 mL ultrapure water, 200 µL solution was added to 0.5 mL ultrafiltration centrifuge tube (molecular weight cut-off: 50,000), centrifuged at 3500 rpm for 30 min, and supernate from the cannula was collected. Another 200 µL solution without centrifugation was set as control. Above experiment was run in triplicate. Drug loading and encapsulation efficiency were calculated according to the following Eqs. (1) and (2):

1 $${\text{Drug Loading}} = \left( {{\text{W}}_{{{\text{Quercetin}}}} /{\text{W}}_{{{\text{QU}} - {\text{Nanogel}}}} } \right){\text{ }} \times 100\%.$$

2 $${\text{Encapsulation Efficiency}} = \left( {{\text{W}}_{{{\text{Quercetin}}}} - {\text{W}}_{{{\text{free Quercetin}}}} /{\text{W}}_{{{\text{QU}} - {\text{Nanogel}}}} } \right){\text{ }} \times 100\% .$$

### Characterization of the nanogel

#### Particle size distribution and zeta potential

The particle size distribution and zeta potential were investigated using a Zetasizer Nano analyzer (Malvern Instruments Ltd., Malvern, UK).

#### Morphology of the nanogel

The morphology of the nanogel was studied using a transmission electron microscope (TEM; H-7650, Hitachi, Japan).

#### Fourier transforming infrared spectrum (FT-IR) analysis

The QU-Nanogel, BLK-Nanogel, quercetin, sodium alginate and physical mixture (quercetin, sodium alginate, CaCl_2_) were dried to constant weight and ground to a powder before using. The FT-IR spectra were recorded using a Bruker Tensor 27 FT-IR spectrometer (Bruker, Germany) in the range of 400–4000 cm^− 1^.

#### X-ray diffraction analysis (XRD)

X-ray diffraction was performed on a D/max-2500 diffractometer (Rigaku, Japan) operated at 50 kV, 180 mA and in the range of 10–50° at a scanning rate of 0.2°/min.

### Antioxidant activities of quercetin and QU-Nanogel

#### DPPH radical scavenging assay

The DPPH solution was prepared in methanol at a concentration of 25 µM. Different concentrations (500 µM, 250 µM, 125 µM, calculated by the concentration of quercetin contained) of quercetin and QU-Nanogel were prepared in methanol and ultrapure water, respectively. The DPPH solution was quickly mixed with quercetin or QU-Nanogel, and the absorbance was measured at a wavelength of 515 nm at 0, 30 and 60 min. The DPPH scavenging was calculated using the following Eq. (3):

3 $${\text{DPPH Scavenging}}\left( \% \right){\text{ }} = \left( {1 - \frac{{A_{{sample}} - ~A_{{blank}} }}{{A_{{max}} }}} \right) \times 100\%$$

where, *A*_*sample*_, *A*_*blank*_ and *A*_*max*_ represent the absorbance of the sample (the quercetin or nanogel with DPPH), mixture (the quercetin or nanogel with methanol), and DPPH solution, respectively (n = 3).

#### Total antioxidant capacity assay (FRAP method)

FRAP (ferric reducing antioxidant power) method was used per the instructions stated on the total antioxidant capacity assay kit. Briefly, different concentrations of quercetin and QU-Nanogel solutions (0.15–1.5 mM, calculated by the concentration of quercetin contained) were prepared in methanol and ultrapure water, respectively. Trolox was served as the positive control. The total antioxidant capacity was represented by the concentration of FeSO_4_ standard solution.

### ALI inducer

PQ is one of the most toxic poisons, has a tend to penetrate the soil and pollute the groundwater resource, leading to a severely toxicity effect to human health. Until now, PQ held the largest share of the underdeveloped regions’ herbicide market. Accidental or intentional self-poisoning with PQ is a major public health issue in some countries, the effective therapy development is urgent. Thus, PQ as the inducer in cell experiment and animal experiments.

### Cell experiments

The human pulmonary carcinoma cell line A549 and expression of GFP in A549 cells (A549-GFP) were obtained from the Cell Culture Center of the Chinese Academy of Medical Sciences (Beijing, China) and cultured by following the SOP prepared in our lab. (The details are shown in Additional file 1: S2 Cell experiment).

### MTS assay

A549 cells were cultured in a 96-well plate at a density of 5 × 10^3^ cells per well and treated with various concentrations of PQ (600 µM, 300 µM, 200 µM, 100 µM), quercetin (5 µM, 2.5 µM), QU-Nanogel (5 µM, 2.5 µM, calculated by the concentration of quercetin contained) and ulinastatin (400 U/mL) and BLK-Nanogel for different incubation times (6 h, 12 h). Except the quercetin was dissolved in 0.1% DMSO, the other drugs were dissolved ultrapure water. Two hours before the end of each time point, MTS reagent (20 µL per well) was added to each well followed by an additional 2 h incubation. The absorbance was recorded at 490 nm using a multifunctional microplate reader (FlexStation3, Molecular Devices, USA).

### Uptake of nanogel

Murine macrophage Raw264.7 cells were used as the cell model for evaluating the uptake of nanogel, Raw264.7 cells were seeded in a 6-well plate at the density of 5 × 10^4^ cells per well, and the RBITC-QU-Nanogel was added for 6 h incubation. The cell nuclei and was stained with DAPI meanwhile the cell membrane was stained with DiO. The uptake of the nanogel by Raw264.7 cells was observed using an inverted fluorescence microscope (OLYMPUS IX73, Japan).

### Protective effects of QU-Nanogel against damage in A549-GFP cells

To test the protective effect of QU-Nanogel and quercetin against PQ poisoning, A549-GFP cells were incubated with 600 µM PQ to induce a damage model. After that, the equimolar concentration of QU-Nanogel and quercetin (5 µM, calculated by the concentration of quercetin contained) were added and incubated for 6 h at 37 °C. The cell nuclei and cell membrane were stained with DAPI and DiI, respectively. The cells survival after QU-Nanogel or quercetin treatment was observed using an inverted fluorescence microscope.

### Determination of the ROS levels

ROS assay has been performed according to the manufacturer’s instructions. After the treatment with 300 µM PQ, A549 cells were incubated with quercetin (5 µM, 2.5 µM), QU-Nanogel (5 µM, 2.5 µM, calculated by the concentration of quercetin contained), BLK-Nanogel and ulinastatin (400 U/ml) at 37 °C for 6 and 12 h, respectively. After drugs treated, cells were incubated with 10 µM DCFH-DA in a 37 °C incubator for 30 min. Finally, fluorescence microscopy was used to observe the ROS production of A549 cells.

### Antioxidative enzyme related mRNA express

The drug administration as the same as ROS assay. After drugs treated, the total RNA was extracted by UNIQ-10 column Trizol total RNA extraction kit. The reverse transcription, Real-time RT-PCR procedure are performed according to our previous research [33] and primer sequences are explained in the Additional file 1: S3 Real-time PCR primer sequences.

### Animal experiment

**Fig. 1:**
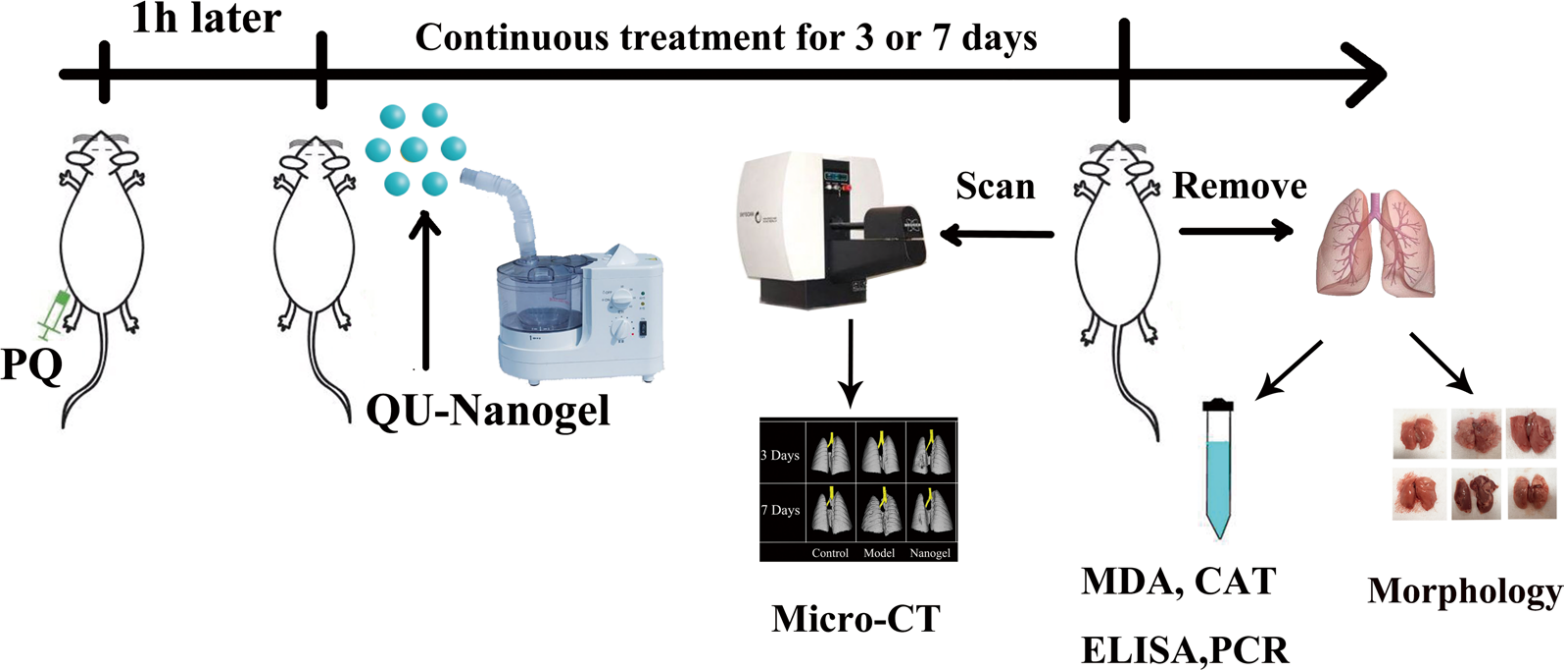
Schematic representation of the executed experiment. The acute lung injury (ALI) rat model was induced by intraperitoneal injection of PQ. After one hour after PQ injection, different drugs were continuously administrated for 3 or 7 days to evaluate the lung protective effects. Inhalation of saline was used as a control, and ulinastatin was used as a positive drug. The micro-CT and photograph of lung tissue sections were performed to assess the lung-protective capacities, and the anti-oxidation and anti-inflammation effects of QU-Nanogel on lung tissue were evaluated by MDA and CAT assays, real-time RT-PCR and ELISA

Health Sprague-Dawley (SD) rats (6–8 weeks, 200 ± 20 g) were purchased from Beijing HFK Bioscience Co., Ltd. The animals were kept at the Experimental Animal Center of Tianjin University of Traditional Chinese Medicine. All the animal experiments were conducted per the set guidelines of the National Institutes of Health (NIH) and the ethics committee of Tianjin University of Traditional Chinese Medicine. They were randomly divided into six groups with six rats in each group: control, model, ulinastatin (60,000 U/kg), QU-Nanogel (50 mg/kg, 100 mg/kg, 150 mg/kg, calculated by the weight of QU-Nanogel), respectively. Five groups of rats were injected PQ (20 mg/kg body weight) intraperitoneally except the control group which was treated with an equal volume of saline. After 1 h, QU-Nanogel groups were inhaled different doses of QU-Nanogel for 20 min and the ulinastatin group was intraperitoneally injected with ulinastatin (60,000 U/kg). The rats were fixed in a self-made bottle that kept air flowing and nose and mouth exposed. The fog outlet of ultrasonic atomizer was connected with rat’s nose by a plastic bellow. The ultrasonic atomizer was set the unified parameters such as fog and air volume. All the rats were sacrificed on day 3 and day 7, after the PQ treatment. Lung tissue was removed carefully and placed on ice packs for taking photos. The left lobes were fixed in 4% formalin and the right lobes were frozen into liquid nitrogen. The process was showed in Fig. 1.

### Tissue distribution of RBITC-QU-Nanogel in rats

The RBITC-QU-Nanogel was administrated by inhalation for 20 min, and then the lung, liver and kidney were observed at predetermined time points (0, 2, 4, 8, 12 and 24 h) using the in vivo fluorescence image system (Kodak FX Pro, Eastman Kodak Company, USA) to get fluorescent images.

### Micro-CT imaging

On day 3 and day 7, the same rat in each group was scanned after different drug treatments, using a micro-CT instrument (Quantum FX, PerkinElmer, USA). All the scans were run for 4.5 min at 360° rotation. All the rats were pre-anesthetized with hydrated chloral, under unconscious state.

### Histopathology

The lung tissue was fixed in 4% neutral formalin for 4 days and embedded in paraffin wax according to standard procedures. Then, 4 μm sections were obtained using a microtome (HM355S, Thermo Scientific, USA). The sections were stained with hematoxylin and eosin (H&E) and imaged under a microscope to study the pathological changes. The alveolar collapse degree was used for scoring. Three high-power visual fields were randomly selected for each section by experienced pathologists with a double-blind method. The lung injury score standard was as follows: 0, normal; 1, extremely mild damage (< 25% area of visual field); 2, mild damage (25–50% area of visual field); 3, moderate damage (50–75% area of visual field); and 4, severe damage (> 75% area of visual field).

### Measurement of malonaldehyde (MDA) and catalase (CAT) in lung tissue

The frozen lung tissue (50 mg) was homogenized in 0.5 mL icy protein lysis. The tissue homogenate was then centrifuged at 3500 rpm at 4 °C for 15 min and the supernate was collected to measure the biochemical index of MDA and CAT according to the specification of the commercial kit, from Beyotime Biotechnology (Shanghai, China).

### Inflammatory cytokines assay

The inflammatory cytokines of Tumor Necrosis Factor-α (TNF-α), Interleukin-6 (IL-6), and Interleukin-1β (IL-1β) were measured with ELISA (Peprotech, Rocky Hill, New Jersey) according to the specification of the commercial kit.

### Real-time RT-PCR for mRNA expression in lung tissue

The total RNA in the lung tissue was extracted using Trizol total RNA extraction kit. The reverse transcription and Real-time PCR experiments were performed according to our previous research (PCR primer sequences are provided in Additional file 1: S3 Real-time PCR primer sequences.

### Statistical analysis

The results were expressed as the mean ± SD for in vitro experiments and expressed as means ± (SE) for animal experiments. The data were analyzed using an Origin 8.0 software (MicroCal, USA). Data was assessed by one-way analysis of variance (ANOVA). The value of *p* < 0.05 or *p* < 0.01 was considered statistically significant. The significance of the main observations and interactions was further investigated using Tukey’s post hoc analyses.

## Results and discussion

For the treatment of lung diseases, direct application of drugs to the lung via aerosol inhalation is widely used due to its high efficacy to the lung and low side effects to normal organs. Nanotechnology also provides new diagnostic and therapeutic options for pulmonary diseases leveraging their unique features for controlled release, reduced drug toxicity, prolonged residence, and targeted delivery [34]. Thus, nanotechnology combined with inhalation therapy is a leading-edge for the treatment of pulmonary disease. The efficiency as well as the inhalation safety assessment of nanomaterials are highly dependent on the particles size, physical properties, and nature of the material [35]. Unlike the inhalable dry powder spray, in this work, the nanogel system in aqueous solution requires a period time to finish inhalation using ultrasonic atomization, rather than a short time of inhaling the large number of powders. In addition, this nanogel system was based on an FDA-approved natural polysaccharide, which has good biodegradability in vivo. Therefore, there is little potential in blocking small blood vessels in the lung.

### Characterization of QU-Nanogel

**Fig. 2:**
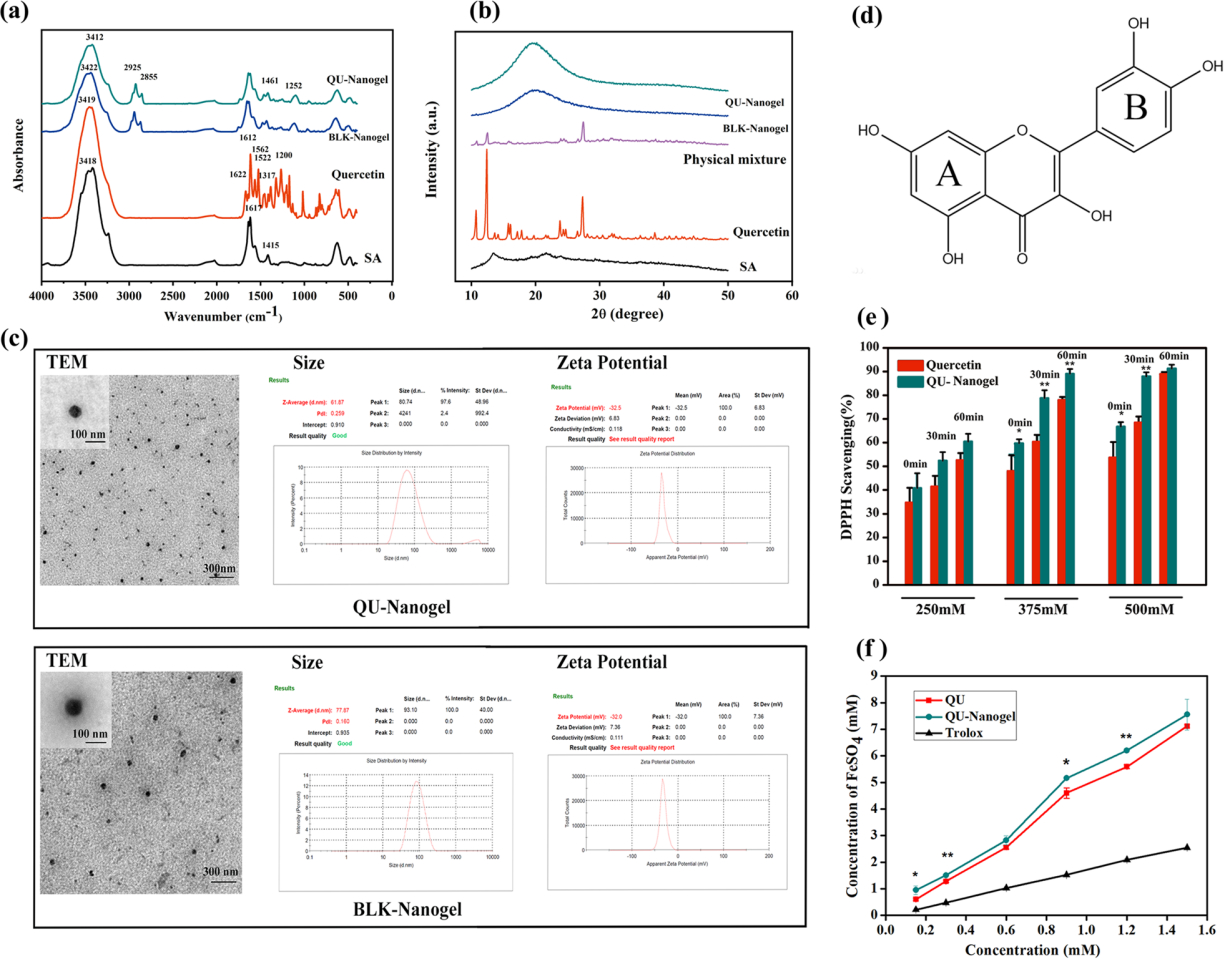
The characterization and antioxidant property of QU-Nanogel. Spectra of QU-Nanogel, BLK-Nanogel, quercetin and sodium alginate (SA) from different tests: (**a**) FT-IR. (**b**) XRD. (**c**) Morphology, size distribution and zeta potential of QU-Nanogel and BLK-Nanogel. Antioxidant activities test of quercetin and QU-Nanogel: (**d**) the chemistry structure formula of quercetin. (**e**) DPPH radical scavenging capacity, (**f**) total antioxidant capacity with FRAP method; (*) denotes the statistical significance of QU-Nanogel compared with quercetin (*p < 0.05; **p < 0.01)

First, the different materials were characterized by FT-IR spectroscopy as shown in Fig. 2a. The spectrum of sodium alginate showed a strong and wide peak at 3418 cm^− 1^. This could be attributed to the stretching vibrations of the –OH groups. The peaks at 1617 cm^− 1^ and 1415 cm^− 1^ can be attributed to the asymmetric and symmetric carboxyl groups (COO–) [36], respectively. In quercetin spectrum, the peak around 3419 cm^− 1^ belonged to the stretching vibration of –OH groups in the aromatic ring. The tiny sharp peaks at 1622 cm^− 1^ and 1200 cm^− 1^ were attributed to the stretching and deformation vibrations of the unsaturated ketone carbonyl group(C=O) [37], respectively. Three sharp peaks at 1612 cm^− 1^, 1562 cm^− 1^, and 1522 cm^− 1^ were the characteristic peaks of the aromatic ring. In nanogels spectra, the peaks at 1617 cm^− 1^ and 1415 cm^− 1^ were lower than sodium alginate, confirming the crosslinking of the COO- groups with Ca^2+^ ions. A new peak near the 1252 cm^− 1^ showed up, and it is attributed to the stretching vibrations of C-O-C structure, which was enhanced due to the crosslinked the C-O-Ca-O-CO- structure as compared to sodium alginate. This could confirm that the alginate could form a more stable three-dimensional structure. After the quercetin was loaded in the nanogel, a broader band peak was observed at 3412 cm^− 1^ compared to free quercetin and BLK-nanogel. The shift to a lower wavenumber was attributed to the hydrogen bonding between the hydroxyl groups of alginate and quercetin.

The crystal structures of the different samples were analyzed by X-ray diffraction analysis as shown in Fig. 2b. The typical tiny peaks of sodium alginate were observed at 13.54° and 23.88° owing to the amorphous structure of sodium alginate [38, 39]. The numerous sharp and strong peaks of quercetin arose at 10.72°, 12.4°, 14.14°, 15.76°, 17.84°, 23.8°, 27.34°, indicating that quercetin has a highly crystalline structure [40]. The XRD spectrum of the physical mixture showed weaker characteristic peaks of quercetin, which could be due to the lower proportion of quercetin the mixture or overlapping peaks of sodium alginate, but was still comparable to the peaks in free quercetin diffraction pattern. In QU-Nanogel groups, most peaks of quercetin had disappeared and a wide diffracted peak could be seen, indicating the presence of a new amorphous structure.

Nanoscale is an important factor for nanogels in biomedical applications. As shown in Fig. 2c, the BLK-Nanogel and QU-Nanogel were nearly spherical in shape as seen under the high-resolution TEM. It was observed that the hydrodynamic average sizes of QU-Nanogel and BLK-Nanogel were 61.87 and 77.87 nm, respectively, in a uniform distribution. Interestingly, the size of the QU-Nanogel was smaller than the blank nanogel under the same preparation conditions. This might be caused by the abundant –OH groups in the quercetin molecule, which could act as a weak cross-linker, causing shrinkage and tightening the nanogel, making the size smaller. The zeta potential of both the nanogels was around − 30 mV, which indicates that both the nanogels possessed good stability. The drug loading and encapsulation efficiency of the “material-drug” nanogel were (0.92 ± 0.02) % and (97.7 ± 1.2) %, respectively.

The antioxidant capacity of different samples was studied using the DPPH method and FRAP method, the DPPH scavenging activities and the ferric reduction ability of equimolar free quercetin monomer and quercetin loaded in QU-Nanogel were compared. In DPPH scavenging activities experiment (Fig. 2e), the QU-Nanogel groups showed a stronger antioxidant capacity than free quercetin at the same concentration. The antioxidant effect of quercetin was attributed to the numbers and position of hydroxyl groups. As shown in Fig. 2d, B ring in basic parent structures of flavonoids is the main reactive site of scavenging free radicals to perform the anti-oxidative effect [41, 42]. The scavenging ability of ortho hydroxyl group is stronger than that of meta hydroxyl groups, which confer upon the quercetin an excellent effect of anti-oxidation, hydrogen atom is donated to DPPH to form a more stable DPPH-H molecule, and quercetin prone to form a resonance stable semiquinone radical structure [43, 44]. The increased antioxidant activity of QU-Nanogel compared to that of free quercetin indicates that, due to a small size, large specific surface area, this “material-drug” structure can also act as a platform with the active sites of the quercetin, which can be exposed to the outlayer like the flagellum, increasing the chance of exposure of the active sites on quercetin. Moreover, the alginate has a certain anti-oxidative effect [45] and the electron cloud density of quercetin molecular was affected during the gel construction process while the activity of hydroxyl atoms in the hydroxyl group was improved, which was beneficial for the hydroxyl group to provide hydrogen atoms to the active free radicals.

FRAP is another method used to determine antioxidant capacity. The principle of FRAP method is that under acidic conditions, the antioxidant can reduce ferric-tripyridyltriazine (Fe^3+^ -tptz) to produce blue Fe^2+^ -tptz, and then the total antioxidant capacity of the sample can be obtained by test the absorbance of blue Fe^2+^ -tptz at the wavelength of 593 nm. As shown in Fig. 2f, the antioxidant properties of QU-Nanogel and quercetin were significantly higher than the positive control group (Trolox), in all the concentration groups of QU-Nanogel showed a stronger antioxidant capacity than free quercetin. It is well-known that flavonoids have strong antioxidant property, and quercetin is one of the most powerful antioxidants in flavonoids. Especially in our prepared novel “drug-alginate” structure, the antioxidant ability of Qu-Nanogel could increase 10% compared to the free quercetin.

### Cell viability and cell uptake of nanogel

**Fig. 3:**
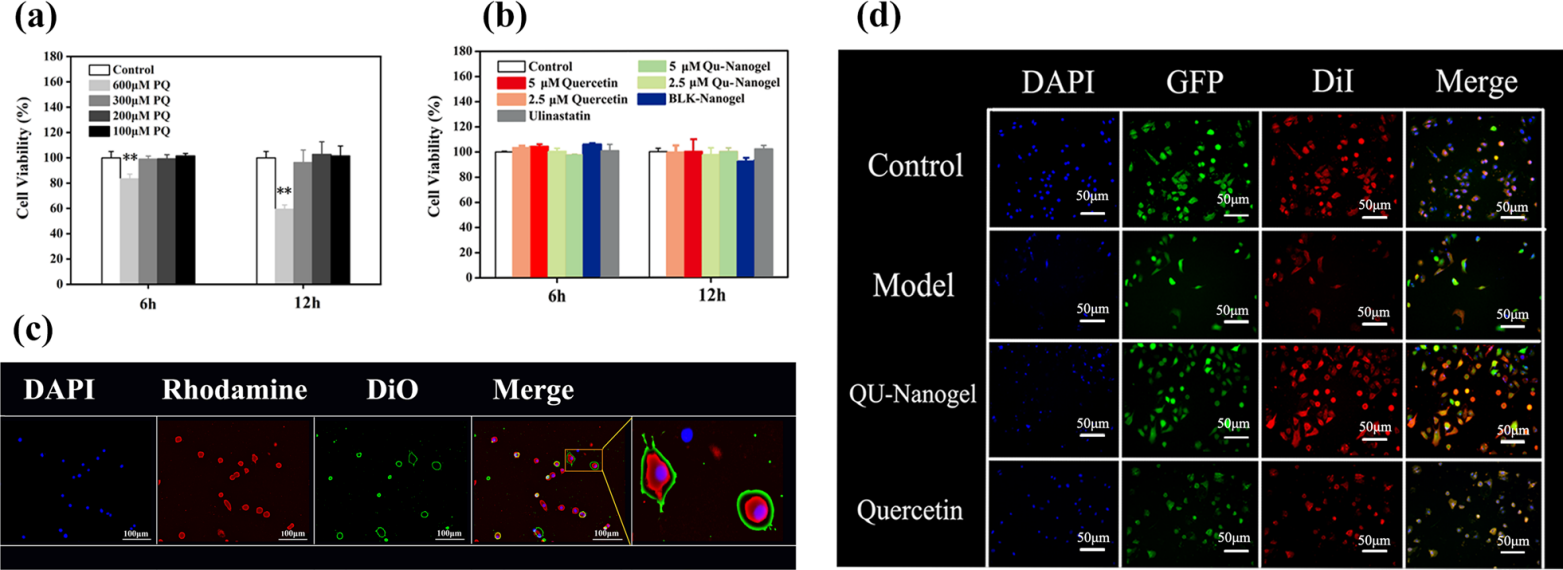
Cell viability, cell uptake and protective effect of QU-Nanogel on A549 cells or RAW264.7 cells. Cell viability of A549 cells treated with different drugs by MTS assay. (**a**) Incubated in different concentrations of PQ for 6 and 12 h. (**b**) Incubated in quercetin, QU-Nanogel, BLK-Nanogel and ulinastatin for 6 and 12 h. (**) denotes statistical significance in comparison to the control group (**p < 0.01). (**c**) The uptake of QU-Nanogel by RAW264.7 cells were analyzed by inverted fluorescence microscope. QU-Nanogel was labeled with rhodamine B isothiocyanate (RBITC) (Red fluorescence), DAPI (Blue fluorescence) and DiO (Green fluorescence) was used to stain the cell nuclei and cell menbrance. (**d**) The protective effects of quercetin and QU-Nanogel on PQ (600 µM) injured A549-GFP cells. The A549-GFP cells emitted bright green fluorescence in the state of cell survival. DiI (Red fluorescence) and DAPI (Blue fluorescence) were used to dye cell membrane and cell nucleus, respectively

Before the evaluation of the nanogel, the cytotoxicity of PQ was tested using the MTS method towards A549 cells as shown in Fig. 3a. Different concentrations of PQ showed different effects on cell viability as compared to the control. A low concentration of 100 µM PQ showed less toxicity towards A549 cells, and 600 µM of PQ caused nearly 40% cell death after 12 h, indicating high toxicity. In this test, PQ was added to create a cell injury model to evaluate the protective effect, and RT-PCR assay was applied to study antioxidant mechanism. In the study of antioxidant mechanism, a sufficient number of cells were needed for RNA extraction, but 600 µM PQ induced more than 40% cells death. However, 300 µM PQ, on one hand, was not affect cell viability; on the other hand, it was high enough to induce oxidant stress for mechanism analysis. Therefore, the concentration of 600 µM PQ was selected for protective effects experiments, and 300 µM PQ was selected for antioxidative enzyme assay (Real-time RT-PCR).

After the experimental concentration of PQ is determined, the cytocompatibility of QU-Nanogel was also evaluated using the MTS method against A549 cells. The cells were cultured in DMEM for 6 and 12 h with different concentrations of the drugs. As shown in Fig. 3b, the A549 cells were highly viable with different drug concentrations. Even in the high concentrations up to 5 µM with 12 h incubation, the viability of cells in QU-Nanogel was above 95%.

Based on the good cytocompatibility of this nanogel, the uptake of nanogel by RAW264.7 cell was investigated by fluorescent images as shown in Fig. 3c. Cellular uptake is a process in which the deformation of the cell membrane transports extracellular biological macromolecules or foreign bodies into the cell. The uptake of RBITC-QU-Nanogel by Raw264.7 cells as observed by a fluorescence microscope. After incubating the Raw264.7 cells with RBITC-QU-Nanogel at 37 °C for 6 h, an intracellular fluorescence was observed in Raw264.7 cells. The first picture shows the nuclei in blue color, and the second picture shows the RBITC-labeled nanogel in red color while the third picture shows the cell membrane in green color. The merged picture and the enlarged image showed that the QU-Nanogel can be taken into the cell after a 6 h incubation period.

### The protective effect of QU-Nanogel in cell damage

The cellular damage model of A549-GFP cell was obtained by incubating with 600 µM PQ for 6 h as shown in Fig. 3d. When the cells were alive, the cells showed green fluorescence under the fluorescence microscope. Once cells died, the A549 cells no longer expressed GFP, and the dead cells showed no fluorescence under the fluorescence microscope. Thus, we can evaluate the protective effect of QU-Nanogel and quercetin against ALI based on the GFP expression. In the model group, the cell survival ratio decreased significantly due to the toxicity of PQ as compared to the other three groups. After adding the quercetin and QU-Nanogel, the cell survival increased, especially in the QU-Nanogel groups, indicating the quercetin loaded nanogel has a stronger protective effect and thus, increasing the cell survival.

### Inhibition of ROS generation by free quercetin and QU-Nanogel

**Fig. 4:**
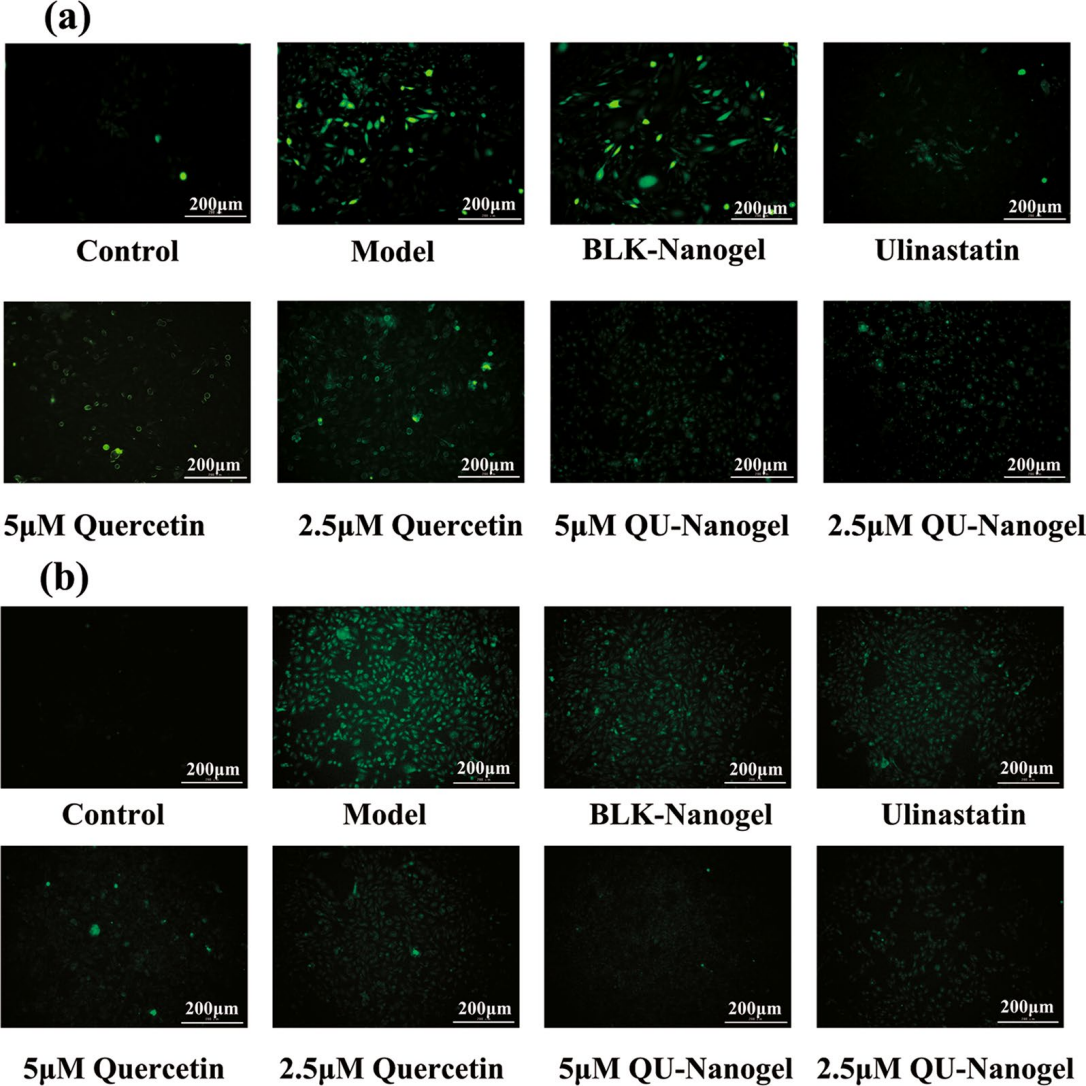
The inhibition of QU-Nanogel on ROS generation. The determination of ROS level was measured by DCFH staining at different time points (**a**) 6 h and (**b**) 12 h. The green fluorescence intensity of DCF represents the ROS level in A549 cells. Quercetin and QU-Nanogel both decreased the ROS level meanwhile QU-Nanogel showed stronger free radical scavenging capacity than quercetin

DCFH-DA can pass through the cell membrane and be hydrolyzed to DCFH under physiological conditions. DCFH is non-fluorescent, but it can be oxidized to fluorescent DCF by ROS [46]. Therefore, the effect of quercetin and QU-Nanogel on inhibiting ROS generation can be determined by its impact on the formation of DCF. As shown in Fig. 4, the ROS accumulation dramatically increased after being treated with PQ for 6 and 12 h. Nevertheless, the production of ROS is reduced after the treatment of ulinastatin or quercetin and QU-Nanogel meanwhile QU-Nanogel showed stronger free radical scavenging capacity than Quercetin, demonstrating that the quercetin and QU-Nanogel may decrease oxidation levels, displayed protective effects through the inhibition of ROS generation.

### Antioxidative enzyme activity of free quercetin and QU-Nanogel

**Fig. 5:**
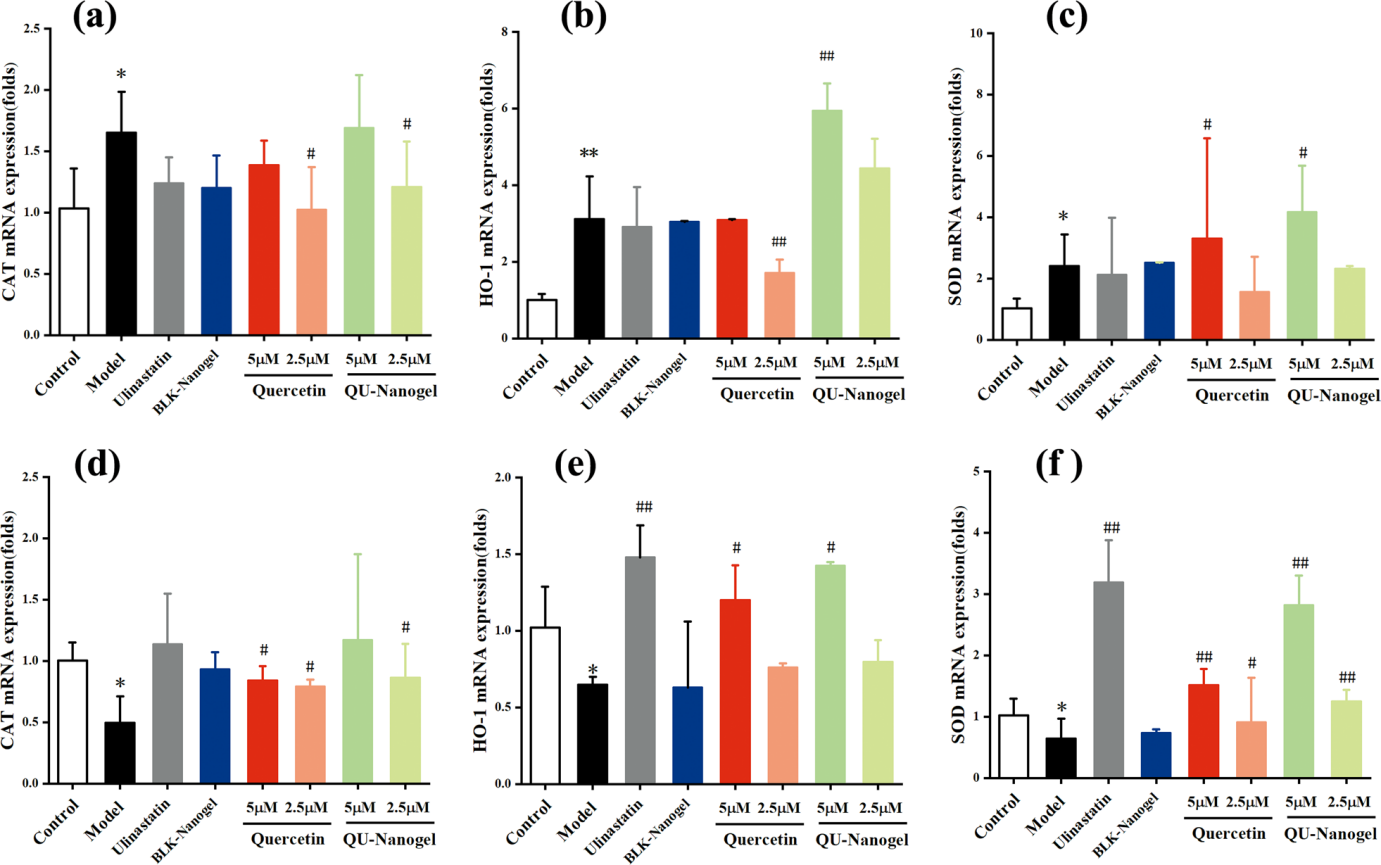
The antioxidative enzyme activity of different drug groups on 300 μM PQ treated A549 cells. The mRNA expression of CAT (**a**, **d**), HO-1 (**b**, **e**) and SOD (**c**, **f**) at 6 and 12 h, respectively; (*) denotes statistical significance compared with the control group (*p < 0.05; **p < 0.01); (^#^) denotes statistical significance compared with the model group (^#^*p* < 0.05; ^##^*p* < 0.01)

In order to further confirm its anti-oxidation capacity, the expression of mRNA of the antioxidant enzyme was tested as shown in Fig. 4. Heme oxygenase-1 (HO-1) is a stress-response protein, the expression of which is transcriptionally regulated by agents that cause oxidative stress [47]. Studies both in vitro and in vivo have shown that HO-1 plays an important role in the protection against cellular and tissue injury mediated by heme and nonheme. Some reports have suggested a possible duality of effects of HO-1 overexpression in oxidative stress. CAT is a stable enzyme scavenger that breaks down hydrogen peroxide into water and oxygen [48]. It is one of the key enzymes in the biological defense system to remove hydrogen peroxide from the body so that cells are free from the toxic effects of H_2_O_2_. Superoxide dismutase (SOD) could protect cells from ROS-mediated damage [49]. This antioxidase system in cellular defenses against oxidative damage. After a 6 h incubation period, the expression of CAT (Fig. 5a), HO-1 (Fig. 5b), and SOD (Fig. 5c) in the model group increased, which could be due to the stress reaction after adding PQ. More of the antioxidant enzymes were needed to resist the oxidation damage, this is the self-protection mechanism of cells. After 12 h incubation, the self-healing ability was reduced, thus decreasing the expression level (Fig. 5d–f). In the ulinastatin group, the expression of all the indicators upgraded. In the quercetin and QU-Nanogel groups, the trend was like the ulinastatin groups, both in the 6 and 12 h, QU-Nanogel and quercetin promote the expression of antioxidase and at the same concentration, QU-Nanogel group showed a higher expression as compared to the free quercetin, especially at a concentration of 5 µM. The expression of antioxidant enzymes was always up-regulated in the nanogel group, suggesting that after the A549 cells were stimulated by oxidative stress, the QU-Nanogel could express related antioxidase genes to form antioxidase in boosting the removal of intracellular oxygen free radicals and oxidative species to resist the oxidant damage.

### Tissue distribution of RBITC-QU-Nanogel in rats after aerosol inhalation

**Fig. 6:**
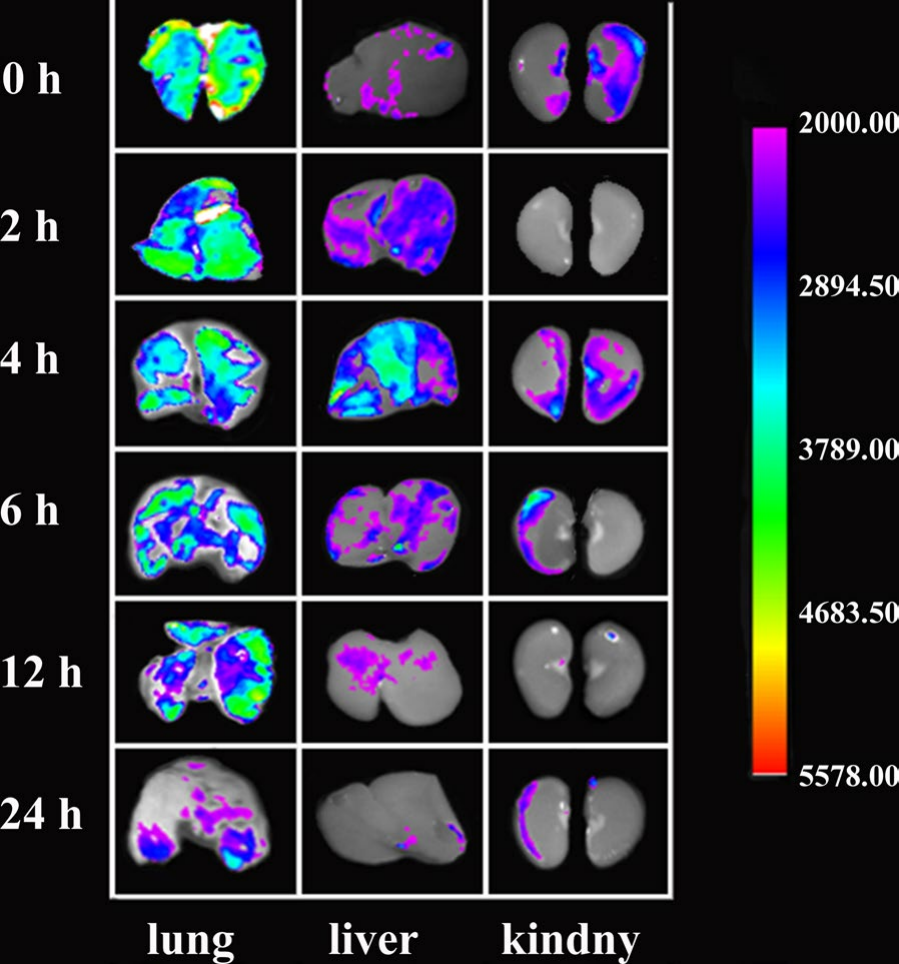
Tissue distribution of RBITC-QU-Nanogel in rats after aerosol inhalation. After aerosol inhalation of RBITC-QU-Nanogel (3 mg/mL) for 20 min, the lung, liver and kidney were observed at predetermined time points (0, 2, 4, 6, 12 and 24 h)

To evaluate QU-Nanogel efficacy and ultrasonic aerosol inhalation on targeting, a rat model of acute lung injury was induced. Firstly, the tissue distribution was studied by fluorescence imaging at different time points. As shown in Fig. 6, after RBITC-QU-Nanogel was inhaled through the respiratory tracts, a strong fluorescence intensity was observed in lung tissue, indicating a good lung target property. As the time extended to 12 h, there was still a relatively strong fluorescence in lung tissue. RBITC fluorescence started to show up in the liver and the kidney as the fluorescence intensity decreased in the lung tissue, indicating nanogel is first absorbed by alveoli, then crossing into the blood circulation and eventually metabolized by the liver and kidney. This might be caused by the small size of the nanogel, therefore it was easy to enter the blood circulation, by the liver and kidney metabolic activity. At 24 h time point, only a weak fluorescence was observed in the three organs which might have been caused by the fluorescence quenching and metabolism of the QU-Nanogel. This result provided evidence that ultrasonic aerosol inhalation is a feasible approach to targeted pulmonary drug delivery and QU-Nanogel is a safe preparation for it can be excreted from the body by the kidney.

### Observation of lung tissue morphology

**Fig. 7:**
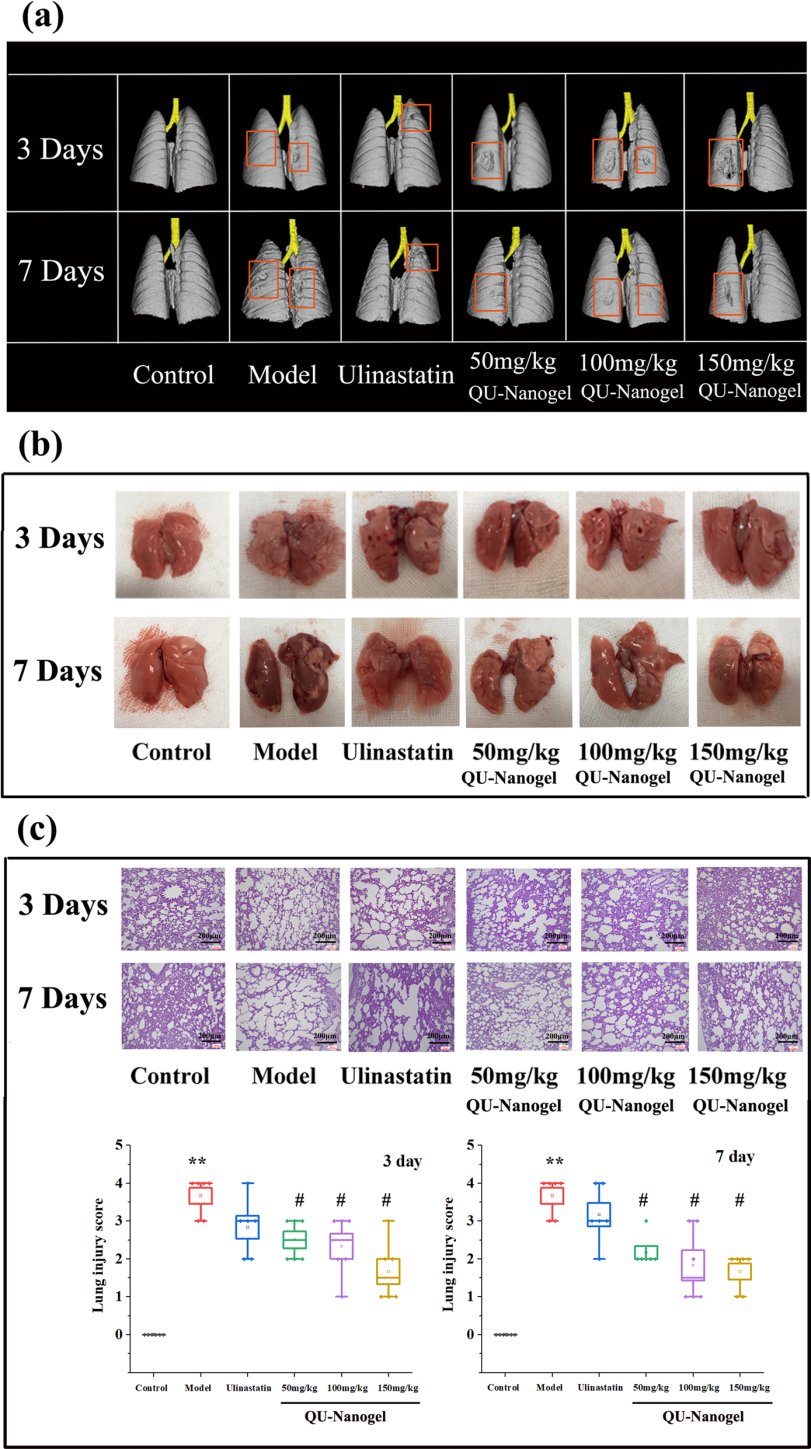
Evaluation of lung-protective capacities of QU-Nanogel on ALI rats. (**a**) The same rat in each group was selected by micro-CT imaging. (**b**) Representative lung tissues separated from rats by anatomic observation. (**c**) H&E staining and score of lung tissue sections. (*) denotes the statistical significance compared with the control group (**p < 0.01); (#) denotes the statistical significance compared with the model group (#*p* < 0.05)

In this part, lung tissue morphology was observed in several ways, including the CT imaging and histopathological method, which have contributed to lung injury research and the therapeutic effects of QU-Nanogel at 3 and 7 days. First, a medical imaging instrument micro-CT scanning and 3D modeling method were used for the lung tissue analysis. As shown in Fig. 7a, the lung tissue showed a complete and smooth surface in the control group. Clearly visible partial defects and shrinkage were observed in the model group at 3 day and 7 day. After 7 day of QU-Nanogel aerosol inhalation, significant recovery of the lung injury was observed, and the injured area decreased. However, it was also observed that the area of lung injury in the model group increased without any drug treatment. Moreover, the lung-protective capacity of this novel mater-drug structural QU-Nanogel was found to be better than the positive drug ulinastatin.

After the continuous tracking by micro-CT, the morphologies of the lung tissues were also evaluated after rats sacrificed as shown in Fig. 7b. In the model group, the lung damages, including bleeding, pulmonary edema and shrinkage could be observed. Moreover, the damage in lung tissue in 7 days was more serious than that in 3 days and changed for deep red. This result was consistent with the micro-CT, indicated that the lung damage is sustained and irreversible. In comparison to this, the control group showed healthy lung tissue illustrated by a pink, smooth and plump surface. After treating with the QU-Nanogel and ulinastatin, the improved symptoms could be observed, especially at high concentrations in the QU-Nanogel group.

Besides micro-CT scanning and anatomic observation, the H&E staining method was also applied for the lung tissue section analysis. As shown in Fig. 7c, pathological changes were studied in different groups. In the control group, healthy alveoli walls connected to each other through tight junctions were observed. However, the alveolar ducts were found to be collapsed in the model group. The alveolar duct collapse was improved significantly after 3 and 7 days of treatment with ulinastatin and QU-Nanogel, especially in the high concentration of the QU-Nanogel group. Lung histological scores in the model group was significantly higher than in the control group, while lung histological scores in the treatment groups were significantly lower than in the model group. These results were consistent with the micro-CT and morphological results.

### Anti-oxidative effects of QU-Nanogel in ALI rats

**Fig. 8:**
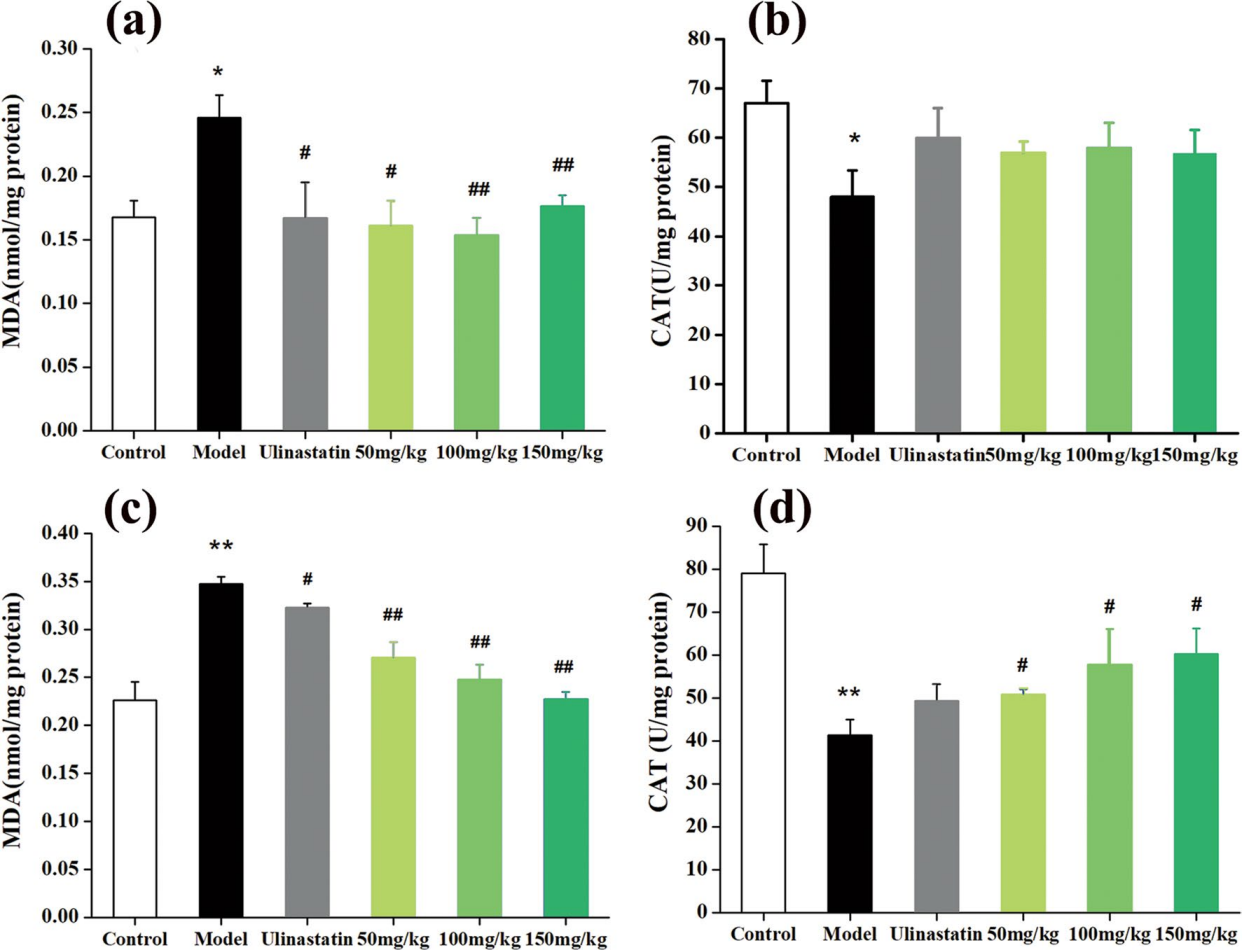
The antioxidative effects of QU-Nanogel on ALI rats. Effects of QU-Nanogel by aerosol inhalation on MDA levels and CAT enzyme activities of rat lung tissue. Changes of MDA level at (**a**) 3 day and (**c**) 7 day (n = 6), changes of CAT activities at (**b**) 3 day and (**d**) 7 day (n = 6), respectively. (*) denotes the statistical significance compared with the control group (*p < 0.05; **p < 0.01); (#) denotes the statistical significance compared with the model group (^#^*p* < 0.05; ^##^*p* < 0.01)

MDA is a low-molecular weight product produced by the decomposition of some primary and secondary lipid peroxidation products, leading to cell metabolism and dysfunction [50]. The content of MDA can reflect the degree of lipid peroxidation and cell damage after free radicals attack the body. CAT is an antioxidant enzyme and the level of enzyme activity represents the level of oxidative damage. As shown in Fig. 7a, b, after 3 days of PQ treatment of the model group, the MDA content increased while CAT activity was decreased. As the time prolonged to 7th day (Fig. 8c, d), the lung injury of QU-Nanogel group was improved significantly, indicating the QU-Nanogel had a better protective effect compared to the positive drug ulinastatin. This protective effect showed a dose-dependent manner. It is noteworthy that, as shown in Fig. 8a, the MDA level in the 150 mg/kg dose group was slightly higher than that of the 50 mg/kg dose group, which might cause by the excessive of QU-Nanogel in a short period time, the higher concentration of QU-Nanogel caused excessive local pressure on the damaged lung tissue. However, the effects changed over time, the therapeutic effect of the high-concentration QU-Nanogel is enhanced and led to corresponding reductions in MDA level.

### Anti-inflammatory effects of QU-Nanogel in ALI rats

**Fig. 9:**
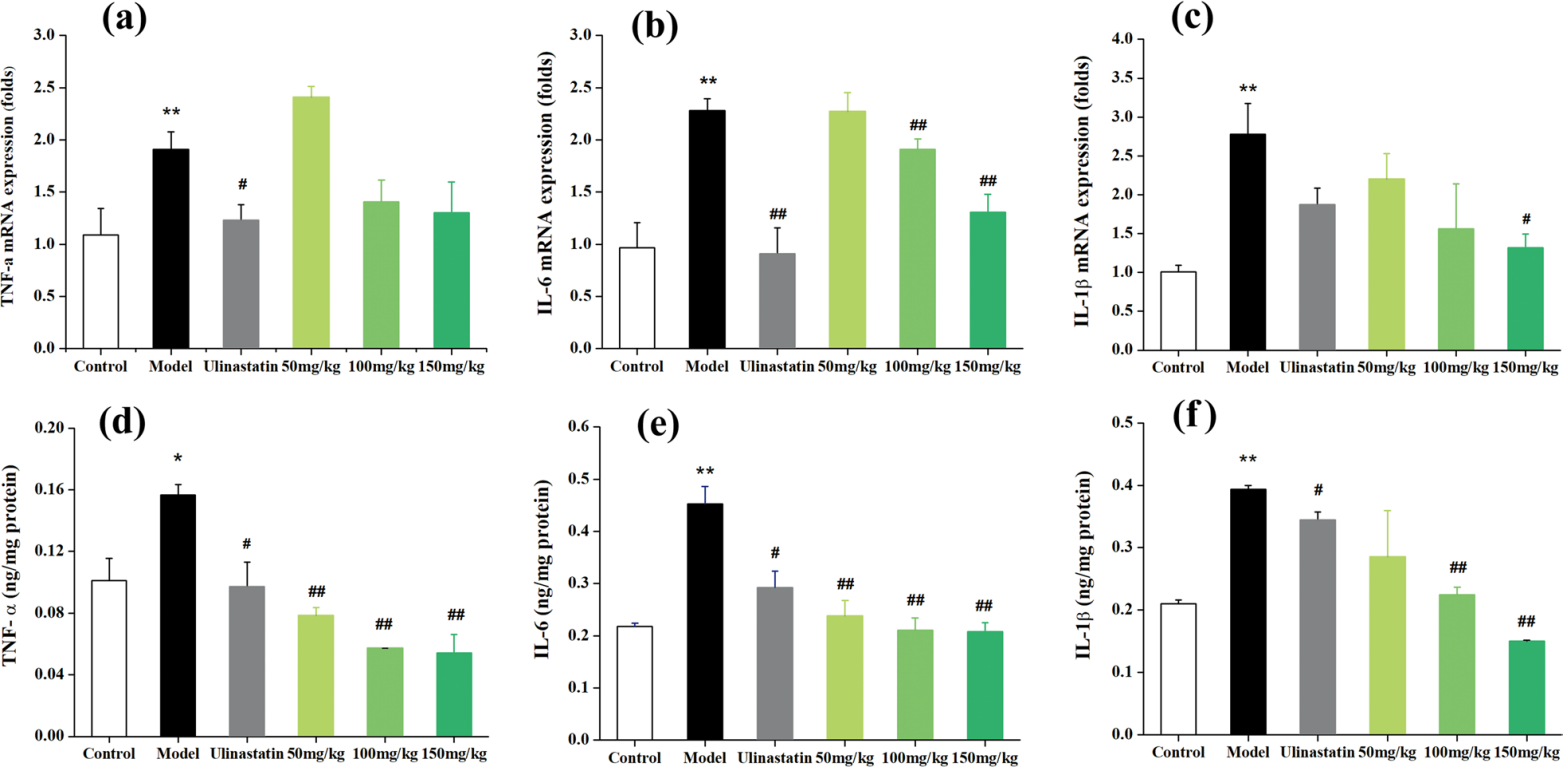
The anti-inflammatory effects of QU-Nanogel on ALI. The rats were exposed by PQ and treated with QU-Nanogel for 7 days. The mRNA expression of TNF-α (**a**), IL-6 (**b**) and IL-1β (**c**) in rat lung tissue (n = 4). The protein expression of TNF-α (**d**), IL-6 (**e**) and IL-1β (**f**) in rat lung tissue (n = 6). (*) denotes the statistical significance compared with the control group (*p < 0.05; **p < 0.01); (#) denotes the statistical significance compared with the model group (^#^*p* < 0.05; ^##^*p* < 0.01)

Acute lung injury is usually accompanied by inflammatory reactions. Inflammatory response is triggered by pro-inflammatory cytokines. TNF-α, IL-6 and IL-1β are known to be required for the induction of acute-phase reactions composed and the primary indicators of inflammation [51]. Thus, the secretions of these three inflammatory cytokines in lung tissue were detected. As shown in Fig. 9a–c, in the model group, the mRNA expression of all the inflammation genes (TNF-α, IL-6, IL-1β) upregulated as compared to the control group. Their expression was significantly decreased after the QU-Nanogel or ulinastatin treatment. Besides the inflammation cytokines detected in the mRNA level, their expressions on protein level were also analyzed (Fig. 9d–f). Compared to the model group, the amount of the indicators was brought down after treating with QU-Nanogel or ulinastatin. After treating with a 150 mg/kg dose of QU-Nanogel, the IL-1β secretion decreased significantly as compared to the positive drug ulinastatin, indicating that QU-Nanogel possessed a better lung protective effect, the trend was found to be similar to the results of real-time RT-PCR assay. Interestingly, a higher expression was observed on the protein level than mRNA level, which might attribute to the self-recovery of the rat at the 7-day time point. The mRNA expression, which precedes protein expression, is already in an adaptation phase as well as gradually insensitive to regulation. The main mechanisms of ALI are known to involve induction of pro-inflammatory cytokines, mediated through activation of nuclear factor-κB (NF-κB). This links inflammatory cytokines to ALI. TNF-α, a ubiquitous pro-inflammatory cytokine, is the first instigator in cell transformation. IL-6 is a pleiotropic cytokine commonly produced at local tissue sites and released into circulation in almost all situations of homeostatic perturbation typically including acute infections [52]. TNF-α stimulates macrophages to produce IL-1, IL-6 and accelerates the extravasation of leukocytes by activating endothelial cells. These cytokines have profound effects on a variety of cell types that, in turn, secrete a variety of inflammatory mediators, forming a complex network of interactions and leading to multiple inflammatory cascades. In the process of ALI, these cytokines leading to recruitment of inflammatory cells into the lung, induced lung damage. Clinical treatment of ALI also includes the administration of anti-inflammatory drugs. Most research have proved that quercetin has a good effect on any pattern of injury. Our results indicated that QU-Nanogel showed a good anti-inflammation property, reduced inflammatory response significantly with a dose-dependent manner, offered protection against ALI.

ALI can be caused by many diseases in the clinic, each ALI patient should be treated personalized due to the difference in etiology and severity and course of disease. PEET, prone positioning and high frequency oscillatory ventilation (HFOV) are the common lung-protective ventilation strategy for achieving lung recruitment and improving oxygenation, however, they also might cause hemodynamic compromise, barotrauma and hypotension [53]. In the drug therapy, corticosteroids could halt the progression to severe and persistent ALI by inhibiting inflammation, pulmonary vasodilator, fibroblast proliferation, and collagen deposition, but long-term stimulation or administration of excessive corticosteroids may lead to protein catabolism, gluconeogenesis and increased the risk of serious neuromuscular weakness and osteoporosis [54]. The classic antioxidant such as glutathione, vitamin C, vitamin E and other drugs such as ulinastatin (a proteinase inhibitor), Chinese medicine injections, showed a curative effect on removing oxygen free radicals and reducing lung injury, but still in the exploratory stages. Overall, the clinical therapy remained primarily anti-inflammation and anti-oxidative. QU-nanogel in this work also based on this strategy as the candidate for treating ALI. QU-nanogel used natural materials or drugs as main ingredients, combined with ultrasonic aerosol inhalation, can reduce the systemic toxicity and side effects in theory, and achieve an efficient therapeutic strategy in ALI.

## Conclusions

In this study, a novel alginate and quercetin based “material-drug” structural inhalable nanogel was prepared. Unlike conventional carriers, in this nanogel, materials and drugs could fix the quercetin in the nanogel by Ca^2+^ and hydrogen bonding, obtaining a “co-construct” water-soluble nanogel system. The QU-Nanogel with a small particle size of less than 100 nm and easier exposes the active site to the surface, thus increasing the chance of active sites reacting with the targets. Furthermore, the bioavailability property of quercetin was improved significantly by QU-Nanogel. QU-Nanogel still showed good physiological activity and could treat ALI rats by acting the down-regulation effects of mRNA and protein expression of inflammation cytokines via ultrasonic aerosol inhalation administration, reducing pulmonary inflammation, thereby preventing the subsequent pulmonary fibrosis. In conclusion, this inhalable QU-Nanogel provides a promising therapeutic strategy for ALI and act as an attractive candidate in vivo.

## Supplementary Information

**Additional file 1.** Condition for HPLC test , Cell experiment and Real-time PCRprimer sequences.

## Abbreviations

ALI: Acute lung injury
CAT: Catalase
MDA: Malonaldehyde
PQ: Paraquat
QU-Nanogel: Quercetin nanogel
SA: Sodium alginate
TNF-α: Tumor necrosis factor-α
IL-6: Interleukin-6
IL-1β: Interleukin-1β
