# Supplementary material for: A novel inhalable quercetin-alginate nanogel as a promising therapy for acute lung injury
Source: J Nanobiotechnology. 2022 Jun 11;20:272. doi: 10.1186/s12951-022-01452-3 (PMC9187928; doi:10.1186/s12951-022-01452-3)
Supplement: Supplementary file 1 — Additional file 1. Condition for HPLC test , Cell experiment and Real-time PCRprimer sequences. [file 12951_2022_1452_MOESM1_ESM.docx]

**Supporting materials**

**A novel inhalable quercetin-alginate nanogel as a promising therapy for acute lung injury**

Yi-Bing Chen, Ya-Bin Zhang, Prabhleen Kaur, Bo-Guang Yang, Yu-Le Wang, Yan Zhu, Lei Ye*, Yuan-Lu Cui*

*^a^ State Key Laboratory of Component-based Chinese Medicine, Research Center of Traditional Chinese Medicine, Tianjin University of Traditional Chinese Medicine, Tianjin 301617, China;*

*^b^ Department of Pharmaceutics, Key Laboratory of Chemical Biology (Ministry of Education), School of Pharmaceutical Sciences, Shandong University, Jinan, Shandong 250012, China;*

*^c^ First Teaching Hospital of Tianjin University of Traditional Chinese Medicine, Tianjin 300381, China;*

*^d^* *National Clinical Research Center for Chinese Medicine Acupuncture and Moxibustion, Tianjin 300381, China;*

*^e^ Shandong Provincial Key Laboratory of Fluorine Chemistry and Chemical Materials, School of Chemistry and Chemical Engineering, University of Jinan, Jinan 250022, China*

*^f^ Research and Development Center of TCM, Tianjin International Joint Academy of Biotechnology & Medicine, TED, Tianjin 300457, China*

*^g^ Department of Chemical Engineering, University of Washington, Seattle, Washington 98195, United States*

*^h^ Department of Biomedical Engineering, The Chinese University of Hong Kong, Hong Kong, China*

*^i^* *Pharmaceutical Informatics Institute, College of Pharmaceutical Sciences, Zhejiang University, Hangzhou, China*

*Corresponding authors:

1.Prof. Yuan-Lu Cui

ORCID: 0000-0003-4392-3713

Research Center of Traditional Chinese Medicine, Tianjin University of Traditional Chinese Medicine, No. 10 Poyanghu Road, West District of Tuanbo new town, Jinghai District, Tianjin, 301617, China

Tel: +86-22-59596229

Fax: +86-22-59596229

E-mail: [cuiyl@tju.edu.cn](mailto:cuiyl@tju.edu.cn)

2. Associate professor: Lei Ye

Department of Pharmaceutics, Key Laboratory of Chemical Biology (Ministry of Education), School of Pharmaceutical Sciences, Shandong University, Jinan, Shandong 250012, China

E-mail address: zbwye@tju.edu.cn (Lei Ye).

**S1. Condition for HPLC test**

Sample procession：The samples are filtered using 0.22 μm organic membrane filtration, then the collected filtrate transfer to the chromatographic sample bottles.

High performance liquid chromatograph: Waters 2695, Milford, MA, USA

Column: Kromasil 100-5 C18; mobile phase: Methanol: water (containing 0.2% Phosphate) = 65:35; Detection wavelength: 360 nm; Flow rate: 1.0 mL/min; Sample: 10 uL, column temperature: 25 °C.

**S2. Cell experiment**

Cell and Cell culture

A549 cells were cultured in DMEM/F12 supplemented with 10 % HI-FBS, 100 U/mL penicillin and 100 μg/mL streptomycin at 37 °C in a fully humidified incubator containing 5 % CO_2_. For all experiments, cells were grown to a confluence of 80–90%, and were subjected to no more than seven cell passages. For real-time RT-PCR, cells were plated at 5×10^5^ cells/ well in 6-well plates for 6 h and 12 h stimulated with various concentrations of Quercetin or QU-Nanogel (5 μM, 2.5 μM), ulinastatin (400U/mL) or BLK-Nanogel after 300 μM PQ treated. Similarly, the control group was given medium alone of equal volume.

**S3. Real-time PCR primer sequences**

Table S1. Real-time PCR primer sequence for animal experiment

| RAT Gene  (Version) | Primer | Sequence (5’-3’) | PCR product (bp) |
| --- | --- | --- | --- |
| GAPDH | Forward | AGACAGCCGCATCTTCTTGT | 142 |
| (NM_017008.4) | Reverse | TGATGGCAACAATGTCCACT |  |
| TNF-α | Forward | TGACCCCCATTACTCTGACC | 142 |
| ((NM_012675.3) | Reverse | CGTGTGTTTCTGAGCATCGT |  |
| IL-1β | Forward | AAAAATGCCTCGTGCTGTCT | 127 |
| (NM_031512.2) | Reverse | GGGATTTTGTCGTTGCTTGT |  |
| IL-6 | Forward | CCGGAGAGGAGACTTCACAG | 134 |
| (NM_012589.2) | Reverse | CAGAATTGCCATTGCACAAC |  |

Table S2. Real-time PCR primer sequence for cell experiment

| Human Gene  (Version) | Primer | Sequence (5’-3’) | PCR product (bp) |
| --- | --- | --- | --- |
| β-actin | Forward | GATGAGATTGGCATGGCTTT | 100 |
| (NM_001101.3) | Reverse | CACCTTCACCGTTCCAGTTT |  |
| CAT | Forward | CGTGCTGAATGAGGAACAGA | 119 |
| (NM_001752.3) | Reverse | AGTCAGGGTGGACCTCAGTG |  |
| SOD | Forward | AGGTCTGAAGGCCTCCATTT | 82 |
| (NM_003102.2) | Reverse | TGGGTGGAAAGGTACCTCAG |  |
| HO-1 | Forward | ATGACACCAAGGACCAGAGC | 153 |
| (NM_002133.2) | Reverse | GTGTAAGGACCCATCGGAGA |  |
